# Neuroprotective Iridoids and Lignans from *Valeriana amurensis*

**DOI:** 10.3390/molecules28155793

**Published:** 2023-08-01

**Authors:** Minhui Ye, Xiaoju Lin, Qiuhong Wang, Bingyou Yang, Changfu Wang

**Affiliations:** 1 Guangdong Engineering Technology Research Center for Standardized Processing of Chinese Materia Medica, School of Chinese Materia Medica, Guangdong Pharmaceutical University, No. 280 Outside Loop East Road of Higher Education Mega Center, Guangzhou 510006, China; 2 Key Laboratory of Basic and Application Research of Beiyao, Heilongjiang University of Chinese Medicine, Ministry of Education, No. 24 HePing Road, Harbin 150040, China

**Keywords:** *Valeriana amurensis*, Aβ_1-42_, PC12 cells, iridoids, lignans, neuroprotective effect

## Abstract

*Valeriana amurensis* (*V. amurensis*) is widely distributed in Northeast China. In addition to medicines, it has also been used to prepare food, wine, tobacco, cosmetics, perfume, and functional foods. Other studies have investigated the neuroprotective effects of *V. amurensis* extract. As the therapeutic basis, the active constituents should be further evaluated. In this paper, six new compounds (**1**–**6**) were isolated, including five iridoids (Xiecaoiridoidside A–E) and one bisepoxylignan (Xiecaolignanside A), as well as six known compounds (**7**–**12**). The neuroprotective effects of **1**–**12** were also investigated with amyloid *β* protein **1**−**42** (Aβ_1-42_)-induced injury to rat pheochromocytoma (PC12) cells. As a result, iridoids **1** and **2** and lignans **6**, **8**, and **9** could markedly maintain the cells’ viability by 3-(4,5)-dimethylthiahiazo (-z-y1)-3,5-di-phenytetrazoliumromide (MTT) and lactate dehydrogenase (LDH) release assay.

## 1. Introduction

The World Alzheimer Report 2018 showed that the number of dementia patients was about 50 million, of whom two-thirds had Alzheimer’s disease (AD). It was expected that this number would increase to 152 million by 2050 [1]. In the past 30 years, some achievements have been made in the development of therapeutic drugs, including acetylcholinesterase inhibitors, glutamate receptor regulators, cerebral circulation improvers, γ-aminobutyric acids, peptides, calcium antagonists, antioxidants, anti-inflammatory medicines, statins, β-amyloid protein vaccines, neurotrophic factors, and central cholinergic receptor agonists [2]. The pathogenesis of AD is extremely complex, and the mechanisms have not been fully revealed. However, the damage or loss of brain neurons is well known in AD patients. Therefore, scholars have increasingly focused on the development of new drugs with brain-neuron-protective activity. Undoubtedly, brain-neuron-protective drugs can prevent the progress of AD to some extent [3]. In the past decades, more than 100 natural products have been considered as potential drugs for the treatment of AD [4,5].

The extracts from *Valeriana* plants can be used to treat nervous system diseases, such as PD, AD, and Huntington’s disease, which are mostly related to brain neuron injuries or apoptosis. Therefore, some scholars have carried out studies on the neuroprotective effects of *Valeriana* in recent years [6]. As a *Valeriana* herb, *Valeriana amurensis* (*V*. *amurensis*) is mainly distributed in Northeast China [7,8]. At present, the roots and rhizomes from *V*. *amurensis* have been used as medicines in China. The extracted essential oil has been well developed and mainly used for preparing food, tobacco, wine, cosmetics, perfume, and so on. As a functional food, *V. amurensis* essential oil is responsible for regulating sleep cycles and helping to fall asleep, such as in the form of Xinjing Valerian Tablets and Valerian Root and Passiflora Compound Nutritional Capsules [6]. In some Chinese books, many healthcare functions of *V. amurensis* have been recorded, including sedative/hypnotic effects, relieving smooth muscle spasms, increasing cardiac blood flow, and reducing myocardial oxygen consumption. However, the development and application of other components from *V. amurensis* has been far from sufficient, and the decoction of *V. amurensis* can only be used as a facial wash and bath softener, leading to a huge waste of resources [7,9]. We have studied its neuroprotective effects against AD previously. The screened neuroprotective fraction from *V*. *amurensis* could protect the brain neurons of an AD mice model from damage [10]. As the therapeutic basis, the active constituents should be revealed. Therefore, a study on the active constituents of *V. amurensis* for protecting the brain’s neurons was carried out. Our study will contribute to making full use of *V. amurensis* resources to increase the economic income and explore some components with healthcare functions.

## 2. Results and Discussion

### 2.1. Structural Elucidation

**Figure 1:**
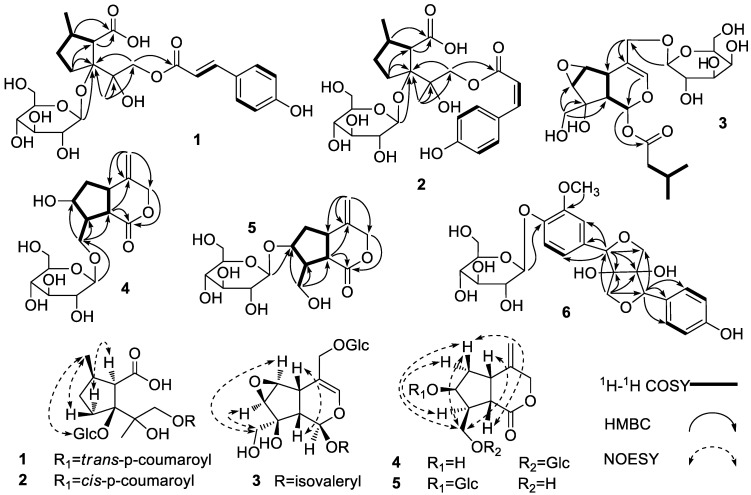
Key ^1^H-^1^H COSY and HMBC correlations of compounds **1**–**6** and NOE correlations of compounds **1**–**5**.

Compound **1** was obtained as a white amorphous powder. The molecular formula of **1** was assigned as C_25_H_34_O_12_ based on the HRESIMS at *m/z* [M-H]^−^ 525.1969 (calcd. 525.1972). The presence of a D-glucose fragment was determined by the hydrolysis experiment of **1**. The ^1^H-NMR spectrum of **1** (Table 1) showed signals of two methyl groups at *δ*_H_ 1.21 (3H, d, *J* = 6.5 Hz, H-9) and 1.47 (3H, s, H-10), respectively. The coupling constant of H-1” (*J* = 7.5 Hz) confirmed the glucopyranosyl moiety as a *β* configuration. The signals at *δ*_H_ 7.57 (1H, d, *J* = 16.0 Hz, H-7′) and 6.28 (1H, d, *J* = 16.0 Hz, H-8′) indicated that an olefinic bond with an *E* configuration was included in **1**, while the typical signals at *δ*_H_ 6.79 (2H, d, *J* = 8.3 Hz, H-3′, 5′) and 7.42 (2H, d, *J* = 8.3 Hz, H-2′, 6′) indicated a p-substituted phenyl group in **1**. Twenty-five carbon signals could be observed in the ^13^C-NMR and DEPT spectra of **1** (Table 2), including six carbon signals of a *β*-D-glucopyranosyl moiety at *δ*_C_ 98.2, 75.1, 78.1, 71.7, 78.2, and 62.8, nine carbon signals of a *trans*-p-coumaroyl group at *δ*_C_ 127.2, 131.4, 117.1, 161.5, 117.1, 131.4, 147.2, 115.0, and 168.6, and a carbon signal of a carboxyl at *δ*_C_ 179.8. The HSQC and ^1^H–^1^H COSY spectra were used to establish two coupling sequences of C(4)/C(5)/C(6)/C(7) and C(6)/C(9) in **1** (Figure 1). The HMBC spectrum was used to further establish the iridomyrmecin-type iridolactone structure of **1** (Figure 1). The HMBC correlations between H-1” and C-3, H-6/H-7 and C-8, CH_3_-10 and C-2, and H-1 and C-9′ suggested that the *β*-D-glucopyranosyl moiety was located at C-3, the carboxyl was located at C-7, the CH_3_-10 was located at C-2, and the *trans*-p-coumaroyl group was located at C-1.

**Figure 2:**
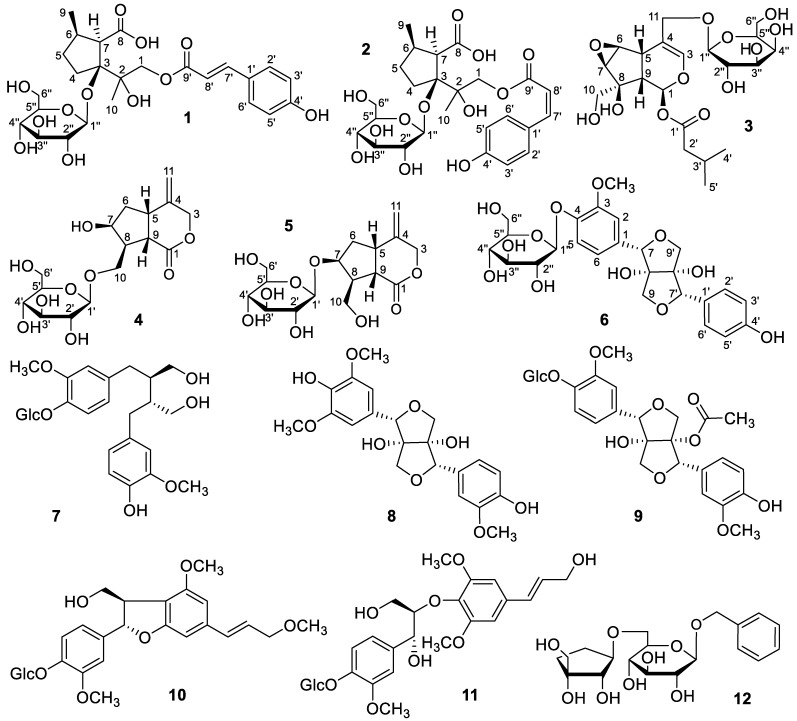
Structures of compounds **1**–**12**.

The NOESY spectrum was used to determine the stereo-configuration of **1** [11]. The NOE correlations between H-7 and H-4α indicated that H-7 was *α*-oriented. The NOE correlations between CH_3_-9 and GlcH-1/H-4β indicated that the glucopyranosyl moiety and CH_3_-9 were *β*-oriented (Figure 1). As a result, the structure of **1** was identified as (7′*E*)-4-p-coumaroyl-2,6*β*-dimethyl-hexahydrocyclopenta-7-carboxylic acid 3-O-*β*-D-glucopyranoside, which was referred to as Xiecaoiridoidside A (Figure 2).

Compound **2** was obtained as a white amorphous powder. The molecular formula of **2** was assigned as C_25_H_34_O_12_ based on the HRESIMS at *m/z* [M-H]^−^ 525.1969 (calcd. 525.1972). The presence of a D-glucose fragment was determined by the hydrolysis experiment of **2**. Most of the ^1^H-NMR and ^13^C-NMR spectral data (Table 1 and Table 2) of **2** were identical to those of **1**. The differences lay in the signals at *δ*_H_ 6.85 (1H, d, *J* = 12.9 Hz, H-7′) and 5.73 (1H, d, *J* = 12.9 Hz, H-8′), which indicated that an olefinic bond with a *Z* configuration was included in **2**. Therefore, the C-4 of **2** was substituted with a *cis*-p-coumaroyl group. The DEPT, HSQC, ^1^H–^1^H COSY, HMBC, and NOESY spectra (Figure 1) were used to identify the structure of **2** as (7′*Z*)-4-p-coumaroyl-2,6*β*-dimethyl-hexahydrocyclopenta-7-carboxylic acid 3-O-*β*-D-glucopyranoside, which was referred to as Xiecaoiridoidside B (Figure 2).

Compound **3** was obtained as a white amorphous powder. The molecular formula of **3** was assigned as C_21_H_32_O_12_ based on the HRESIMS at *m/z* [M + Na]^+^ 499.1787 (calcd. 499.1791). The presence of a D-galactose fragment was determined by the hydrolysis experiment of **3**. The ^1^H-NMR spectrum of **3** (Table 1) showed signals of two methyl groups at *δ*_H_ 0.94 (6H, d, *J* = 6.7 Hz, CH_3_-4′, 5′). The coupling constant of H-1” (*J* = 8.1 Hz) confirmed the galactopyranosyl moiety as a *β* configuration. Twenty-one carbon signals could be observed in the ^13^C-NMR and DEPT spectra of **3** (Table 2), including six carbon signals of a *β*-D-galactopyranosyl moiety at *δ*_C_ 100.4, 72.6, 73.2, 69.2, 75.6, and 63.4, and five carbon signals of an isovaleryl group at *δ*_C_ 173.2, 44.3, 27.0, 22.7, and 22.7. The HSQC and ^1^H–^1^H COSY spectra were used to establish two coupling sequences of C(9)/C(5)/C(6)/C(7) and C(2′)/C(3′)/(4′)/C(5′) in **3** (Figure 1). The HMBC spectrum was used to establish the iridoid skeleton of **3** (Figure 1). In addition, the H-1” correlated with C-11, H-10 correlated with C-7, C-8, and C-9, and H-1 correlated with C-1′ suggested that the *β*-D-galactopyranosyl moiety was linked to C-11, hydroxymethyl was linked to C-8, and the -O-isovaleryl group was linked to C-1, respectively.

The NOESY spectrum was used to determine the stereo-configuration of **3**. The H-5 and H-9 were *β*-oriented based on the biogenetic of the iridoid [12]. The NOE correlations between H-6 and H-10, and between H-5 and H-9, but the absence of correlations between H-5 and H-10, H-5 and H-7, H-5 and H-1, H-9 and H-6, and H-9 and H-7, indicated that H-1, H-6, H-7, and 8-CH_2_OH were *α*-oriented, while 8-OH, the 1-O-isovaleryl group, and the 6,7-epoxy group were *β*-oriented (Figure 1). As a result, the structure of **3** was identified as 8*β*,10-dihydroxy-6*β*,7*β*-epoxy-11-*β*-D-galactopyranosyl-1-isovaleryl-iridoid, which was referred to as Xiecaoiridoidside C (Figure 2).

Compound **4** was obtained as a white amorphous powder. The molecular formula of **4** was assigned as C_16_H_24_O_9_ based on the HRESIMS at *m/z* [M + Na]^+^ 383.1314 (calcd. 383.1318). The presence of a D-glucose fragment was determined by the hydrolysis experiment of **4**. The ^1^H-NMR spectrum of **4** (Table 1) showed signals of a terminal olefinic bond at *δ*_H_ 5.08 (1H, s, H-11a) and 5.20 (1H, s, H-11b). The coupling constant of H-1′ (*J* = 7.8 Hz) confirmed the glucopyranosyl moiety as a *β* configuration. Sixteen carbon signals could be observed in the ^13^C-NMR and DEPT spectra of **4** (Table 2), including six carbon signals of a *β*-D-glucopyranosyl moiety at *δ*_C_ 104.9, 75.3, 78.3, 71.7, 78.2, and 62.8. The HSQC and ^1^H–^1^H COSY spectra were used to establish two coupling sequences of C(5)/C(6)/C(7)/C(8)/C(9) and C(8)/C(10) in **4** (Figure 1). The HMBC spectrum was used to establish the iridoid skeleton of **4** (Figure 1). The HMBC correlations between H-1′ and C-10 suggested that the *β*-D-glucopyranosyl moiety was linked to C-10.

The NOESY spectrum was used to determine the stereo-configuration of **4 [13]**. The NOE correlations between H-5 and H-10, H-10 and H-9, H-6*α* and H-8, H-6*β* and H-10, and H-6*β* and H-9, but the absence of correlations between H-9 and H-7, and between H-5 and H-7, indicated that H-7 and H-8 were *α*-oriented, while 7-OH and 8-CH_2_OGlc were *β*-oriented (Figure 1). As a result, the structure of **4** was identified as 7*β*-hydroxy-4-methylenehexahydrocyclo-penta[*c*]pyran-1(3*H*)-one 10-*O*-*β*-D-glucopyranoside, which was referred to as Xiecaoiridoidside D (Figure 2).

Compound **5** was obtained as a white amorphous powder. The molecular formula of **5** was assigned as C_16_H_24_O_9_ based on the HRESIMS at *m/z* [M + Na]^+^ 383.1314 (calcd. 383.1318). The presence of a D-glucose fragment was determined by the hydrolysis experiment of **5**. Most of the ^1^H-NMR and ^13^C-NMR spectral data (Table 1 and Table 2) of **5** were similar to those of **4**. The differences lay in the signals at *δ*_H_ 3.86 (2H, d, *J* = 6.8 Hz, H-10), *δ*_C_ 83.7 (C-7), and 61.7 (C-10), which indicated that the *β*-D-glucopyranosyl moiety was linked to C-7 in **5**. The DEPT, HSQC, ^1^H–^1^H COSY, HMBC, and NOESY spectra (Figure 1) were used to identify the structure of **5** as 8*β*-hydroxymethyl-4-methylenehexahydrocyclo-penta[*c*]pyran-1(3*H*)-one 7-*O*-*β*-D-glucopyranoside, which was referred to as Xiecaoiridoidside E (Figure 2).

Compound **6** was obtained as a white amorphous powder. The molecular formula of **6** was assigned as C_25_H_30_O_12_ based on the HRESIMS at *m/z* [M + Na]^+^ 545.1631 (calcd. 545.1635). The presence of a D-glucose fragment was determined by the hydrolysis experiment of **6**. The ^1^H-NMR spectrum of **6** (Table 1) showed a signal of a methoxyl group at *δ*_H_ 3.88 (3H, s, 3-OCH_3_). The coupling constant of H-1” (*J* = 7.1 Hz) confirmed the glucopyranosyl moiety as a *β* configuration. Moreover, the ^1^H-NMR spectrum of **6** also showed the signals of a 1,4-disubstituted phenyl group and a 1,3,4-trisubstituted phenyl group. Twenty-five carbon signals could be observed in the ^13^C-NMR and DEPT spectra of **6** (Table 2), including six carbon signals of a *β*-D-glucopyranosyl moiety, twelve carbon signals of two phenyl groups, and a methoxyl group, as well as two oxygenated quaternary groups, two oxygenated methines, and two oxygenated methylenes. These data show typical characteristics of bisepoxylignans. The HSQC and ^1^H–^1^H COSY spectra further confirmed the coupling sequences of two phenyl groups in **6** (Figure 1). The HMBC spectrum was used to establish the bisepoxylignan structure of **6** (Figure 1), and the correlation between H-1” and C-4 suggested that the *β*-D-glucopyranosyl moiety was located at C-4.

The specific rotation of **6** was determined as [*α*]$\frac{20}{D}$ − 49.6° (*c* 0.10, MeOH), and a negative Cotton effect was observed at λ_max_ = 235 nm, which was similar to that of (7*R*,8*S*,7′*R*,8′*S*)-5-methoxyprinsepiol-4-O-*β*-D-glucopyranoside reported in reference [14]. Moreover, both of their ^1^H-NMR and ^13^C-NMR data at positions C-7, C-8, and C-9 were almost identical. Therefore, the absolute configuration of **6** was the same as that of (7*R*,8*S*,7′*R*,8′*S*)-5-methoxyprinsepiol-4-O-*β*-D-glucopyranoside. As a result, the structure of **6** was identified as (7*R*,8*S*,7′*R*,8′*S*)-3′-demethoxy-prinsepiol-4-O-*β*-D-glucopyranoside, which was referred to as Xiecaolignanside A (Figure 2).

The known compounds were identified as (-)-secoisolariciresinol 4-O-*β*-D-glucopyranoside (**7**) [15], (7*R*,8*S*,7’*R*,8’*S*)-5-methoxyprinsepiol (**8**) [14], 1-acetoxypinoresinol-4′-*β*-glucoside (**9**) [16], dehydrodiconiferyl alcohol 9’-methyl ether-4-O-*β*-D-glucoside (**10**) [17], citrusin B (**11**) [18], and icariside F2 (**12**) [19] by comparing their NMR spectroscopic and physical data with reported values.

### 2.2. Detection of the Neuroprotective Effects

**Figure 3:**
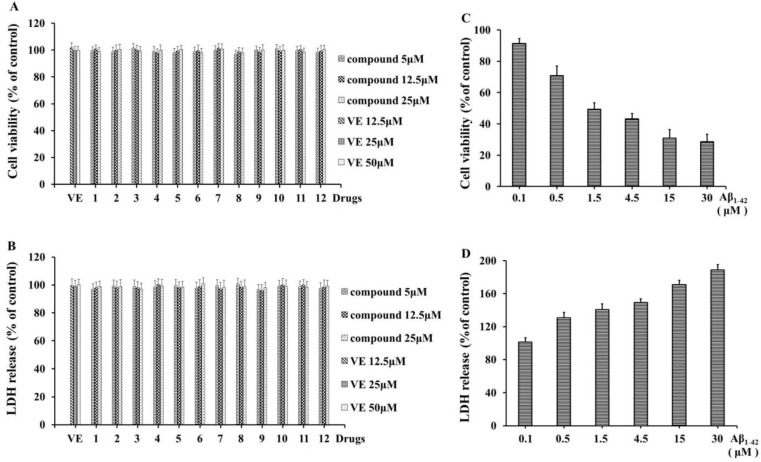
(**A**) MTT assay for the effects of different concentrations (5, 12.5, and 25 μM) of compounds **1**–**12** on the viability of PC12 cells. Vitamin E (VE) was the positive drug. (**B**) Colorimetric LDH assay kit testing the effects of different concentrations (5, 12.5, and 25 μM) of compounds **1**–**12** on the LDH release of PC12 cells. (**C**) MTT assay for the effects of different concentrations (0.1, 0.5, 1.5, 4.5, 15, and 30 μM) of Aβ_1–42_ on the cell viability of PC12 cells. (**D**) Colorimetric LDH assay kit testing the effects of different concentrations (0.1, 0.5, 1.5, 4.5, 15, and 30 μM) of Aβ_1–42_ on the LDH release of PC12 cells. All data were recorded as means ± S.D. (% of control) from three independent experiments, and the control was the normal PC12 cells untreated with any other drugs.

As we all know, the amyloid β protein (Aβ) plays a key role in AD and can be a potential therapeutic target. Therefore, all isolated compounds (**1**–**12**) were evaluated for their neuroprotective activity against the Aβ_1-42_-induced death of rat pheochromocytoma (PC12) cells by 3-(4,5)-dimethylthiahiazo (-z-y1)-3,5-di-phenytetrazoliumromide (MTT) and lactate dehydrogenase (LDH) assay. As a result, no significant influence was observed in PC12 cells treated with different concentrations (5, 12.5, and 25 μM) of compounds **1**–**12** for 24 h (Figure 3A,B). PC12 cells were pretreated with different concentrations (0.1, 0.5, 1.5, 4.5, 15, and 30 μM) of Aβ_1-42_ for 20 h, which induced decreases in cell viability from 91% to 28% in a concentration-dependent manner (Figure 3C). Similarly, Aβ_1-42_ caused the release of LDH to increase from 101% to 188% (Figure 3D). Therefore, 1.5 μM Aβ_1-42_-induced cell viability of 49.45% and LDH release of 140.62% was used as model group.

**Figure 4:**
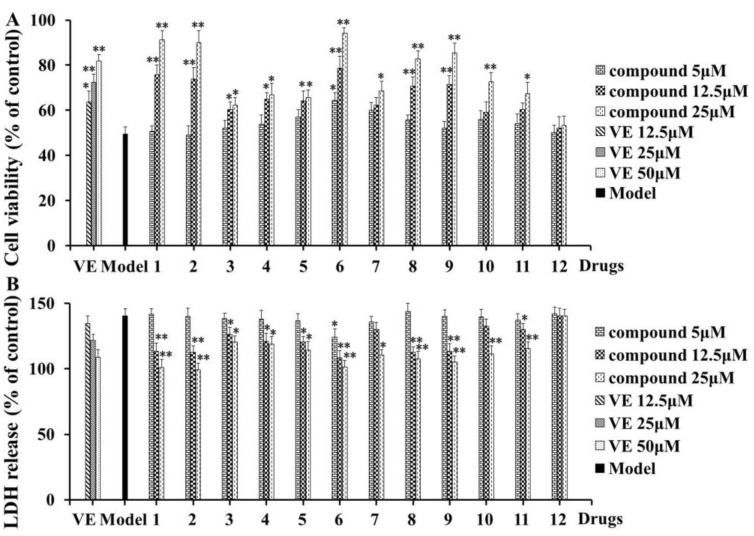
The effects of compounds **1**–**12** on model PC12 cells induced by Aβ_1–42_. The cell viability (**A**) and LDH release (**B**) from three independent experiments are expressed as the mean ± SD (*n* = 9). VE was the positive drug. ** Significant difference compared with model (** *p* < 0.01). * Significant difference compared with model (* *p* < 0.05).

To determine the protective effects of compounds **1**–**12** against Aβ_1-42_-induced neurotoxicity, PC12 cells were treated with 1.5 μM Aβ_1-42_ for 20 h after pretreatment with compounds **1**–**12** at concentrations of 5, 12.5, and 25 μM for 4 h. As shown in Figure 4A,B, the viability of cells treated with iridoids **1** and **2** was markedly increased in a dose-independent manner compared to that of the model group, and the same was true for lignans **6**, **8**, and **9**. This result was further confirmed by the reduction in LDH release from cells treated with compounds **1**, **2**, **6**, **8**, and **9**, especially at the concentration of 25 μM. Iridoids and lignans are the main components of *V*. *amurensis*. The above results adequately elucidate the neuroprotective effect of *V*. *amurensis* extracts on AD model mice.

## 3. Materials and Methods

### 3.1. General Experimental Procedures

The extracts of *V*. *amurensis* were separated by column chromatography (CC) of macroporous resin (AB-8 crosslinked polystyrene, Nan Kai, Tianjin, China), silica gel (200–300 mesh, Haiyang Chemical Group Co. Ltd., Qingdao, China), and ODS-A (120A, 50 mm; YMC, Kyoto, Japan), successively. Preparative HPLC: a Waters 2535 instrument in tandem with a Waters Sunfire prep C18 OBD^TM^ 10 μm (19 × 250 mmi.d.) column for preparing compounds, and detection with UV-2998 and RI-2414 detectors. The NMR spectra were assayed on a Bruker DPX 400 instrument (Bruker SpectroSpin, Karlsruhe, Germany). A Xero Q Tof MS spectrometer (Waters, Milford, MA, USA) was used to measure the HRESIMS. A Shimadzu FTIR-8400S (Kyoto, Japan) was used to determine the IR spectra of the isolated compounds. The sugar derivatives from isolated compounds were analyzed on GC-MS (Agilent, California, CA, USA). PC12 cells were bought from the Institute of Biochemistry and Cell Biology (Shanghai, China). The PC12 cells were grown in DMEM (HyClone, NRH0020) with a 1% antibiotic mixture of penicillin–streptomycin and 5% fetal bovine serum in an atmosphere of 5% CO_2_ at 37 °C. A bicinchoninic acid (BCA) protein assay kit (Nianjing Jiancheng Bioengineering Institute, Nanjing, China) was used to determine the total protein concentrations of the PC12 cells. The colorimetric LDH level was assayed with a colorimetric LDH assay kit on a microplate reader (VICTOR^TM^ ×3, PerkinElmer, Inc., Waltham, MA, USA).

### 3.2. Plant Materials

The roots and rhizomes of *V. amurensis* were collected from the Great Xing’an Mountains area (Heilongjiang province, China) and identified by Zhenyue Wang, who is a pharmacognosy professor at Heilongjiang University of Chinese Medicine. The voucher specimen (No. 20200913) was deposited at the Herbarium of Heilongjiang University of Chinese Medicine, Harbin, China.

### 3.3. Extraction and Isolation

The neuroprotective fraction of *V. amurensis* was prepared according to the method reported previously [10]. Briefly, 8.0 kg of dried roots and rhizomes was extracted with 75% EtOH (64 L) under reflux three times (for 1.5 h each), and the 75% EtOH extract (1437.6 g) was obtained after removing the solvent. Then, petroleum ether (5 × 10 L) was used to partition the 75% EtOH extract in water to obtain the water extract (1044.5 g). The water extract was subjected to an AB-8 macroporous resin column (10 × 60 cm) and eluted with H_2_O and 50% EtOH successively to obtain the 50% EtOH fraction (230.8 g), which was the neuroprotective fraction in the previous study. The extract of the neuroprotective fraction (180.0 g) was separated via normal column chromatography (CC) with silica gel, eluted with CH_2_Cl_2_–MeOH (from 20:1 to 1:1, *v*/*v*), and fractions I–VII were obtained. Fraction II (27.4 g) was subjected to silica gel CC and eluted with CH_2_Cl_2_–MeOH (from 30:1 to 10:1, *v*/*v*) to obtain sub-fractions II_1_–II_5_. Fraction II_2_ (3.8 g) was further chromatographed over silica gel, eluted with CH_2_Cl_2_–MeOH (15:1, *v*/*v*), and compound **8** (96.3%, 45 mg) was precipitated from MeOH directly. Fraction II_4_ (11.2 g) was further separated by ODS CC and eluted with MeOH–H_2_O (10–50% gradient) to give two fractions. Preparative HPLC (CH_3_CN–H_2_O, 8 mL/min) was used to separate and purify the two fractions, and compounds **1** (95.2%, 32 mg, *t*_R_ = 38.5 min, 33% CH_3_CN), **2** (96.6%, 21 mg, *t*_R_ = 38 min, 33% CH_3_CN), **10** (98.5%, 46 mg, *t*_R_ = 32 min, 28% CH_3_CN), and **9** (96.2%, 31 mg, *t*_R_ = 29 min, 28% CH_3_CN) were obtained. Fraction IV (20.4 g) was subjected to silica gel CC and eluted with CH_2_Cl_2_–MeOH (from 20:1 to 10:1, *v*/*v*) to obtain sub-fractions IV_1_–IV_3_. Fraction IV_2_ (5.6 g) was further separated by ODS CC and eluted with MeOH–H_2_O (10–50% gradient) to give two fractions. Preparative HPLC (CH_3_CN–H_2_O) was used to separate and purify the two fractions, and compounds **3** (97.6%, 37 mg, *t*_R_ = 58 min, 15% CH_3_CN), **7** (98.0%, 25 mg, *t*_R_ = 31.0 min, 17% CH_3_CN), **11** (98.5%, 35 mg, *t*_R_ = 27 min, 17% CH_3_CN), **12** (96.3%, 44 mg, *t*_R_ = 22 min, 17% CH_3_CN), and **6** (98.2%, 48 mg, *t*_R_ = 14 min, 17% CH_3_CN) were obtained. Fraction VI (32.3 g) was separated by ODS CC and eluted with MeOH–H_2_O (10–40% gradient) to give sub-fractions VI_1_-VI_4_. Fraction VI_3_ (8.7 g) was separated and purified with preparative HPLC (CH_3_CN–H_2_O) to obtain compounds **4** (97.1%, 35 mg, *t*_R_ = 36 min, 5% CH_3_CN) and **5** (96.8%, 29 mg, *t*_R_ = 37.0 min, 5% CH_3_CN)

#### 3.3.1. Xiecaoiridoidside A (**1**)

White amorphous powder, [*α*]$\frac{20}{D}$ − 15.4° (*c* = 0.10, MeOH); IR (KBr) *ν*_max_ 3431, 3376, 2956, 2923, 2855, 1768, 1725, 1612, 1462, 1208, 1105, 1074, 945, 840 cm^−1^; ESIMS *m*/*z* 509 (100) [M + H]^+^; HRESIMS [M + H]^+^*m*/*z* 509.2019, calcd. 509.2023 for C_25_H_32_O_11_H; ^1^H- and ^13^C-NMR data; see Table 1 and Table 2.

#### 3.3.2. Xiecaoiridoidside B (**2**)

White amorphous powder, [*α*]$\frac{20}{D}$ − 14.3° (*c* = 0.10, MeOH); IR (KBr) *ν*_max_ 3431, 3376, 2956, 2923, 2855, 1768, 1725, 1612, 1462, 1204, 1105, 1074, 832 cm^−1^; cm^−1^; ESIMS *m*/*z* 509 (100) [M + H]^+^; HRESIMS [M + H]^+^*m*/*z* 509.2019, calcd. 509.2023 for C_25_H_32_O_11_H; ^1^H- and ^13^C-NMR data; see Table 1 and Table 2.

#### 3.3.3. Xiecaoiridoidside C (**3**)

White amorphous powder, [*α*]$\frac{20}{D}$ − 24.6° (*c* = 0.10, MeOH); IR (KBr) *ν*_max_ 3431, 3376, 2925, 2874, 1745, 1455, 1254, 1064, 885 cm^−1^; ESIMS *m*/*z* 499 (100) [M + H]^+^; HRESIMS [M + Na]^+^ *m*/*z* 499.1787 calcd. 499.1791 for C_21_H_32_O_12_H; ^1^H- and ^13^C-NMR data; see Table 1 and Table 2.

#### 3.3.4. Xiecaoiridoidside D (**4**)

White amorphous powder, [*α*]$\frac{20}{D}$ − 45.8° (*c* = 0.10, MeOH); IR (KBr) *ν*_max_ 3454, 3314, 2925, 2852, 1755, 1630, 1136, 895 cm^−1^; ESIMS *m*/*z* 383 (100) [M + Na]^+^; HRESIMS [M + Na]^+^ *m*/*z* 383.1314 calcd. 383.1318 for C_16_H_24_O_9_H; ^1^H- and ^13^C-NMR data; see Table 1 and Table 2.

#### 3.3.5. Xiecaoiridoidside E (**5**)

White amorphous powder, [*α*]$\frac{20}{D}$ − 52.3° (*c* = 0.10, MeOH); IR (KBr) *ν*_max_ IR (KBr) *ν*_max_ 3454, 3316, 2925, 2852, 1755, 1630, 1150, 895 cm^−1^; ESIMS *m*/*z* 383 (100) [M + Na]^+^; HRESIMS [M + Na]^+^ *m*/*z* 383.1314 calcd. 383.1318 for C_16_H_24_O_9_H; ^1^H- and ^13^C-NMR data; see Table 1 and Table 2.

#### 3.3.6. Xiecaolignanside A (**6**)

White amorphous powder, [*α*]$\frac{20}{D}$ − 49.6° (*c* = 0.10, MeOH); *ν*_max_ 3454, 1705, 1665, 1512, 1423, 1358, 1228, 1090, 905 cm^−1^; ESIMS *m*/*z* 545 (100) [M + Na]^+^; HRESIMS [M + Na]^+^ *m*/*z* 545.1631 calcd. 545.1635 for C_25_H_30_O_12_H; ^1^H- and ^13^C-NMR data; see Table 1 and Table 2.

### 3.4. Monosaccharide Analysis of **1**–**6**

Acid hydrolysis of compounds **1***–***6** was conducted in accordance with the method reported in reference [20], with some differences. Briefly, the monosaccharides were obtained from hydrolyzing compounds **1***–***6** (2.5 mg of each) with 2.0 mL of H_2_SO_4_ (2 mol/L). The monosaccharides were further treated with trimethylchlorosilane to obtain the sugar derivatives of **1***–***6**. The sugar derivatives were analyzed by GC-MS, and the monosaccharide of compounds **1**, **2**, and **4**–**6** was determined to be D-glucose (*t*_R_ = 11.45 min). The monosaccharide of compound **3** was determined to be D-galactose (*t*_R_ = 8.35 min).

### 3.5. Determination of the Cells’ Viability

The culture and treatment of PC12 cells were similar to the method reported previously in [21]. Briefly, the PC12 cells were cultured in 6-well plates (6 × 10^5^ cells/well) for 24 h; after that, different concentrations (5, 12.5, and 25 μM) of compounds **1**–**12** were added to incubate for 24 h, while different concentrations (0.1, 0.5, 1.5, 4.5, 15, and 30 μM) of Aβ_1–42_ were added to incubate for 20 h. Then, the effects of compounds **1**–**12** on normal cells and a suitable concentration of Aβ_1–42_ for inducing PC12 cells were confirmed by MTT and intracellular LDH release assay. The cell viability ratios of each group to the normal cell group (control) were calculated and recorded.

The culture and treatment of cells were prepared as described above. Then, compounds **1**–**12** at concentrations of 5, 12.5, and 25 μM were added to incubate for 4 h, while the control and model groups were added with equal volumes of medium. As an effective drug in a previous study, vitamin E (VE) was used as a positive control [22]. Neurotoxicity was induced in all groups of cells by 1.5 μM for 20 h, except for the control group. Then, 20 μL MTT solutions (5 mg/mL) were added to each well, and the cells were incubated at 37 °C for another 4 h. We next aspirated off the supernatants and then dissolved formazan crystals with DMSO. The microplate reader was used to measure the optical density of each well at 490 nm. Cell viability was also detected by measuring the LDH released [21]. A 50 μL culture supernatant was collected from each well for detecting the LDH activity (U/L) with a colorimetric LDH assay kit, according to the manufacturer’s instructions. Colorimetric absorbance was measured on a plate reader at 570 nm. Assays of MTT and LDH were all repeated in three independent experiments, with six wells for each, and the results were expressed as a percentage of the control group, whose optical density was set at 100%.

### 3.6. Statistical Analysis

All data are presented as the mean ± SD. One-way analysis of variance (ANOVA) was used to perform statistical comparisons, and differences with *p* values < 0.05 according to the *t*-test were considered to be significant.

## 4. Conclusions

In conclusion, six new compounds (Xiecaoiridoidside A–E (**1**–**5**) and xiecaolignanside A (**6**)) and six known compounds (**7**–**12**) were isolated from the roots and rhizomes of *V. amurensis*. The chemical structures of Xiecaoiridoidside A–E and xiecaolignanside A were identified by the analyses of their 1D and 2D NMR, HRESIMS, and other spectra. In addition, the neuroprotective effects of all isolated compounds were also investigated with an Aβ_1-42_-induced PC12 cell injury model, and iridoids **1** and **2**, as well as lignans **6**, **8**, and **9**, could markedly maintain the cells’ viability. Therefore, some iridoids and lignans are likely responsible for the neuroprotective effects of *V. amurensis*.

## Figures and Tables

**Table 1:** ^1^H NMR data of compounds **1**–**6** (400 MHz in CD_3_OD at 30 °C; *δ* in ppm; *J* in Hz).

Position	1	2	3	4	5	6
**1**	4.26, d (11.8) 4.81, d (11.8)	4.23, d (11.8) 4.79, d (11.8)	6.40, brs			
**2**						7.11, brs
**3**			6.397, brs	4.71, d (11.9) 4.75, d (11.9)	4.67, d (11.7) 4.75, d (11.7)	
**4**	1.69, m 2.32, m	1.69, m 2.32, m				
**5**	1.48, m 1.96, m	1.48, m 1.96, m	3.09, brd (8.5)	3.47, m	3.49, m	7.15, d (8.3)
**6**	2.28, m	2.28, m	4.04, d (2.0)	1.68, m 2.17, dd (13.2, 7.5)	1.62, m 2.52, dd (13.6, 7.1)	6.95, brd (7.6)
**7**	2.94, d (4.5)	2.89, d (4.5)	3.37, d (2.2)	4.41, t (3.7)	4.38, t (3.7)	5.01, s
**8**				2.59, m	2.56, m	
**9**	1.21, d (6.5)	1.20, d (6.5)	2.05, m	2.96, t (10.0)	2.99, dd (9.1, 11.2)	4.12, d (9.4) 3.99, d (9.4)
**10**	1.47, s	1.44, s	3.69, d (3.2)	4.15, dd (5.7, 9.6) 3.79, t (9.6)	3.86, d (6.8)	
**11**			4.21, d (11.6) 4.35, d (11.6)	5.08, s 5.20, s	5.08, s 5.19, s	
**1′**				4.33, d (7.8)	4.43, d (7.7)	
**2′**	7.42, d (8.3)	7.62, d (8.3)	2.18, d (6.7)	3.20, t (8.5)	3.18, t (8.5)	7.25, d (8.3)
**3′**	6.79, d (8.3)	6.74, d (8.3)	2.02, m	3.37, m	3.36, m	6.78, d (8.4)
**4′**			0.94, d (6.7)	3.30, m	3.29, m	
**5′**	6.79, d (8.3)	6.74, d (8.3)	0.94, d (6.7)	3.28, m	3.29, m	6.78, d (8.4)
**6′**	7.42, d (8.3)	7.62, d (8.3)		3.68, dd (4.6, 12.0) 3.86, brd (11.8)	3.68, dd (3.6, 11.8) 3.86, brd (11.3)	7.25, d (8.3)
**7′**	7.57, d (16.0)	6.85, d (12.9)				4.97, s
**8′**	6.28, d (16.0)	5.73, d (12.9)				
**9′**						3.97, d (9.4) 4.10, d (9.4)
**3-OCH_3_**						3.88, s
**1″**	4.41, d (7.5)	4.41, d (7.5)	4.72, d (8.1)			4.90, d (7.1)
**2″**	3.20, t (8.0)	3.20, t (8.0)	3.35, m			3.50, m
**3″**	3.39, m	3.39, m	4.05, m			3.40, m
**4″**	3.32, m	3.32, m	3.48, dd (2.7, 9.3)			3.41, m
**5″**	3.30, m	3.30, m	3.68, m			3.48, m
**6″**	3.88, brd (11.6) 3.67, brd (11.9)	3.88, brd (11.6) 3.67, brd (11.9)	3.66, m 3.86, brd (9.8)			3.70, brd (13.3) 3.87, m

**Table 2:** ^13^C-NMR data of compounds **1**–**6** (100 MHz in CD_3_OD at 30 °C; *δ* in ppm).

Position	1	2	3	4	5	6
1	68.7, CH_2_	68.3, CH_2_	90.8, CH	177.2, C	177.3, C	133.4, C
**2**	87.9, C	87.7, C				113.8, CH
**3**	94.6, C	94.7, C	142.4, CH	72.6, CH_2_	72.8, CH_2_	150.6, C
**4**	31.7, CH_2_	31.7, CH_2_	109.8, C	144.2, C	144.1, C	147.8, C
**5**	34.4, CH_2_	34.4, CH_2_	35.4, CH	40.9, CH	41.1, CH	117.7, CH
**6**	39.5, CH	39.4, CH	59.9, CH	41.1, CH_2_	40.2, CH_2_	121.6, CH
**7**	61.6, CH	61.3, CH	60.3, CH	72.9, CH	83.7, CH	88.9, CH
**8**	179.8, C	179.7, C	80.2, C	50.5, CH	51.8, CH	89.1, C
**9**	22.0, CH_3_	22.0, CH_3_	43.6, CH	45.2, CH	45.1, CH	76.8, CH_2_
**10**	18.0, CH_3_	18.1, CH_3_	67.2, CH_2_	69.6, CH_2_	61.7, CH_2_	
**11**			69.8, CH_2_	113.9, CH_2_	114.0, CH_2_	
**1′**	127.2, C	127.6, C	173.2, C	104.9, CH	105.6, CH	129.1, C
**2′**	131.4, CH	134.0, CH	44.3, CH_2_	75.3, CH	75.6, CH	130.3, CH
**3′**	117.1, CH	116.12, CH	27.0, CH	78.3, CH	78.3, CH	115.9, CH
**4′**	161.5, C	160.3, C	22.7, CH_3_	71.7, CH	71.7, CH	158.5, C
**5′**	117.1, CH	116.12, CH	22.7, CH_3_	78.2, CH	78.2, CH	115.9, CH
**6′**	131.4, CH	134.0, CH		62.8, CH_2_	62.8, CH_2_	130.3, CH
**7′**	147.2, CH	146.1, CH				89.1, CH
**8′**	115.0, CH	116.09, CH				89.4, C
**9′**	168.6, C	167.6, C				77.1, CH_2_
**3-OCH_3_**						56.9, CH_3_
**1″**	98.2, CH	98.2, CH	100.4, CH			103.1, CH
**2″**	75.1, CH	75.1, CH	72.6, CH			75.1, CH
**3″**	78.1, CH	78.1, CH	73.2, CH			78.3, CH
**4″**	71.7, CH	71.7, CH	69.2, CH			71.5, CH
**5″**	78.2, CH	78.2, CH	75.6, CH			78.0, CH
**6″**	62.8, CH_2_	62.8, CH_2_	63.4, CH_2_			62.7, CH_2_

## Data Availability

Data are contained within the article.

## Sample Availability

Samples of compounds **1–6** are available from the authors.
